# Tuberculosis can cause anything in the world except pregnancy!

**DOI:** 10.1186/1749-8090-10-S1-A233

**Published:** 2015-12-16

**Authors:** Vasudev B Pai, Ganesh S Kamath, Sambhram Shetty, Nitin T Patil

**Affiliations:** 1 Department of CVTS, Kasturba Medical College, Manipal University, Manipal, Karnataka, 576104, India; 2 Department of Anaesthesia, Kasturba Medical College, Manipal University, Manipal, Karnataka, 576104, India

## Background/Introduction

Tuberculosis has been reported to cause aortic aneurysms and coronary aneurysms.

## Aims/Objectives

To describe a case report with a surprise diagnosis of tuberculosis.

## Method

We present a patient with dialysis dependant end stage renal disease for AR with a dilated root and chest wall mass with coronary aneurysms needing aortic valve replacement and coronary artery bypass grafting whose final diagnosis was tuberculosis.

## Results

A 37 year old male patient presented with class III dyspnoea on exertion. He was hypertensive with dialysis dependent end stage renal disease and dialysed using an AV Fistula on the left upper limb. Echocardiography showed severe AR and LV dysfunction. CT Coronary angiography showed aortic root to be 5 cm and saccular aneurysms in the circumflex and the right coronary artery with complete occlusion of RCA. A Bentall procedure was planned. Intraoperatively, there was a mass arising from anterior chest wall measuring 6 cm × 5 cm attached to the Left Internal Mammary artery with multiple enlarged paratracheal and mediastinal lymph nodes. Frozen section from the mass and lymph nodes did not reveal any malignancy. The oncosurgeon felt that the mass was malignant looking at its extent and CT characteristics. Pragmatically a mechanical aortic valve replacement and saphenous vein grafts to the OM and PDA were performed after a discussion between the clinicians instead of a root replacement. The final histopathology showed tuberculosis of the lymph nodes with no malignancy in the mass. The patient underwent dialysis postoperatively and needed inotropes till day 4. He was started on anti-tuberculosis medications and was discharged on postoperative day 10. In retrospect the coronary aneurysms and the root dilatation with aortic regurgitation could have been caused by tuberculosis.

## Discussion/Conclusion

Surprises are still possible in today's world even with the best investigations and as surgeons we have to make difficult decisions on table.

## Consent

Written informed consent was obtained from the patient's next of kin for publication of this abstract and any accompanying images. A copy of the written consent is available for review by the Editor of this journal.

